# Optimizing placebo and minimizing nocebo effects through communication: e-learning and virtual reality training development

**DOI:** 10.1186/s12909-024-05671-0

**Published:** 2024-07-01

**Authors:** Janine Westendorp, Liesbeth M. van Vliet, Stefanie H. Meeuwis, Tim C. olde Hartman, Ariëtte R. J. Sanders, Eric Jutten, Monique Dirven, Kaya J. Peerdeman, Andrea W. M. Evers

**Affiliations:** 1 Health, Medical and Neuropsychology Unit, Institute of Psychology, Leiden University, Wassenaarseweg 52, 2333 AK Leiden, The Netherlands; 2 Center for Interdisciplinary Placebo Studies (IPS) Leiden, Leiden, The Netherlands; 3 Department of Primary and Community Care, Radboud University Medical Center, Nijmegen, The Netherlands; 4 General Medical Practice Van Lennep Huisartsenpraktijk, Driebergen, The Netherlands; 5 The Simulation Crew (TSC), Nijmegen, The Netherlands; 6 Dutch Institute for Rational Use of Medicine (IVM), Utrecht, The Netherlands

**Keywords:** Placebo effect, Nocebo effect, Communication training, Virtual reality, e-learning

## Abstract

**Background:**

The effects of many treatments in healthcare are determined by factors other than the treatment itself. Patients’ expectations and the relationship with their healthcare provider can significantly affect treatment outcomes and thereby play a major role in eliciting placebo and nocebo effects. We aim to develop and evaluate an innovative communication training, consisting of an e-learning and virtual reality (VR) training, for healthcare providers across all disciplines, to optimize placebo and minimize nocebo effects through healthcare provider-patient communication. The current paper describes the development, mid-term evaluation, optimization, and final evaluation of the communication training, conducted in The Netherlands.

**Methods:**

The development of both the e-learning and the VR training consisted of four phases: 1) content and technical development, 2) mid-term evaluation by healthcare providers and placebo/communication researchers, 3) optimization of the training, and 4) final evaluation by healthcare providers. To ensure the success, applicability, authenticity, and user-friendliness of the communication training, there was ongoing structural collaboration with healthcare providers as future end users, experts in the field of placebo/communication research, and educational experts in all phases.

**Results:**

Placebo/communication researchers and healthcare providers evaluated the e-learning positively (overall 7.9 on 0–10 scale) and the content was perceived as useful, accessible, and interesting. The VR training was assessed with an overall 6.9 (0–10 scale) and was evaluated as user-friendly and a safe method for practicing communication skills. Although there were some concerns regarding the authenticity of the VR training (i.e. to what extent the virtual patient reacts like a real patient), placebo and communication researchers, as well as healthcare providers, recognized the significant potential of the VR training for the future.

**Conclusions:**

We have developed an innovative and user-friendly communication training, consisting of an e-learning and VR training (2D and 3D), that can be used to teach healthcare providers how to optimize placebo effects and minimize nocebo effects through healthcare provider-patient communication. Future studies can work on improved authenticity, translate the training into other languages and cultures, expand with additional VR cases, and measure the expected effects on providers communication skills and subsequently patient outcomes.

## Background

The effects of many regular clinical treatments in healthcare are partially determined by factors other than the treatment itself [1, 2]. Patients’ expectations and the relationship with their healthcare provider can significantly affect treatment outcomes and thereby play a major role in placebo and nocebo effects [3]. We define placebo and nocebo effects as the changes in patient outcomes that can be explained by the expectations someone has about the treatment[4]. The underlying biopsychosocial processes involved in placebo and nocebo effects have been extensively studied. These processes include learning mechanisms (e.g. patients’ previous experiences or clinicians’ suggestions) and the healthcare provider-patient relationship (e.g. emphatic behavior) that can influence patient expectations and trust [3, 5–8]. As the healthcare provider-patient interaction plays such an important role in eliciting placebo and nocebo effects [9–12], training healthcare providers’ communication with their patients is pivotal for optimizing healthcare.

Experts in placebo research consented that there are several strategies to optimize placebo effects and minimize nocebo effects through communication in clinical practice [4, 13]. For example, healthcare providers could enhance treatment effects if they outline the expected benefits from treatment [14], prevent side effects by fine-tuning the information they give to patients [15–17], and increase trust and satisfaction through an empathetic attitude [18–21]. However, experts also agree that these communication strategies are currently underutilized, and that healthcare providers should preferably be trained to address placebo and nocebo effects via their communication [13].

Our goal was to develop and evaluate an innovative communication training for healthcare providers to optimize placebo and minimize nocebo effects through healthcare provider-patient communication. We aimed for the training to be suitable for healthcare providers across disciplines at every level, whether they are actively practicing or still in training, thus ensuring its broad applicability. The communication training will exist of two advanced eHealth components: an e-learning and virtual reality (VR) training. Using these eHealth techniques has the potential for great outreach as it can be easily offered online. Other advantages over hiring teachers or actors are: costs-efficiency, standardized teaching and practicing, safe learning environment, and opportunities for extensive repetitive practice [22–25]. Additionally, the use of virtual patients yields comparable learning effects compared to role-playing actors [26, 27]. The aim of the communication training was threefold: 1) to familiarize healthcare providers with state-of-the art knowledge on placebo and nocebo effects, 2) to raise awareness about the role of placebo and nocebo effects in everyday clinical practice, and 3) to teach communication techniques that can optimize placebo effects and minimize nocebo effects in clinical practice. The current paper describes the development, mid-term evaluation, optimization, and final evaluation of the communication training.

## Methods

The content of the communication training was based on the most recent scientific insights and expert consensus on placebo and nocebo effects, which has been investigated systematically during the first [4] and second [13] official Society for Interdisciplinary Placebo Studies (SIPS) conferences in 2017 and 2019. The training consists of two parts. First, the background theory, empirical evidence and communication skills are taught in an e-learning. Second, hands-on practice is offered in a VR training. Both the e-learning and the VR tool were developed in Dutch.

The e-learning was developed first and its content was the starting point for the VR training. The development of both the e-learning and the VR training took place between May 2021 and October 2022 and was divided into four phases: 1) content and technical development, 2) mid-term evaluation by healthcare providers and placebo/communication researchers, 3) optimization of the training, and 4) final evaluation by healthcare providers. To ensure the success, applicability, authenticity, and user-friendliness of the training, in all phases there was ongoing structural collaboration with a group of experts. This group consisted of all authors and the experts mentioned in the acknowledgements, in total including two general practitioners, two anesthesia practitioners (one physician and one physician assistant), one VR expert (and his team members) who developed the VR application, one educational expert (and her team members) who developed the e-learning, and fifteen national and international researchers (most with backgrounds in biomedical and health sciences, some of whom are also working in clinical practice). The authors together set up the content and design of the training. Throughout the phases, updates were consistently shared with the other experts for feedback and approval. The studies were conducted in The Netherlands and approved by the Ethical Committee of Psychology Research of Leiden University (2022–03-01-A.W.M. Evers-V2-3783 and 2022–06-10-A.W.M. Evers-V2-4051).

### E-Learning development and evaluation

#### Content determination

**Fig. 1:**
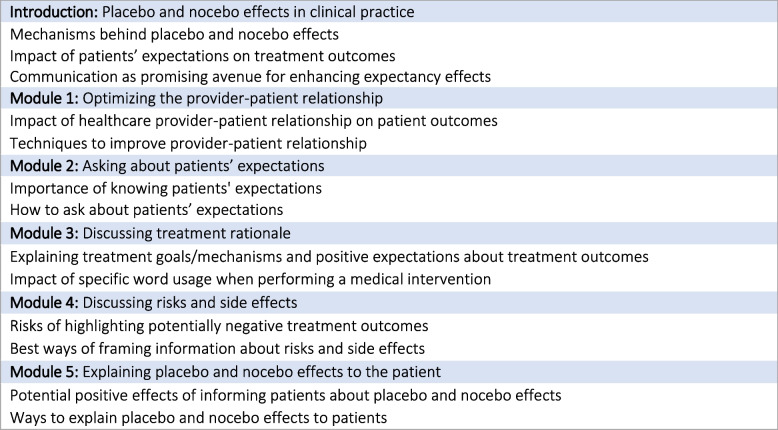
Overview of the e-learning’s main structure and contents

For the development of the e-learning we collaborated with a non-profit medical education provider, the Dutch Institute for Rational Use of Medicine (IVM). To determine the specific design and content topics of the e-learning, a brainstorm session was organized with an expert group of national and international clinicians and placebo/communication researchers (i.e. all authors and experts mentioned in acknowledgements). Subsequently, a content framework was created in collaboration with an education developer from IVM, which was sent to the expert group for approval. All involved experts agreed on the topics to be included (Fig. 1).

#### E-learning structure

The e-learning structure is based on leading didactic theories [28–31]. To activate and motivate, the e-learning starts with a welcome video, followed by an audio message from a general practitioner (AS) who already makes extensive use of the communication techniques. Second, healthcare providers are challenged to think about their own knowledge and skills, and what they want to improve. Third, an introduction about placebo and nocebo effects in clinical practice is given. This introduction is followed by five substantive modules (Fig. 1). Each module contains a video, which focuses on background knowledge, and textual information, which focuses on practical skills. Subsequently, an assignment is given (‘step-by-step case’) in which the healthcare provider can practice the learned techniques on an own (imaginary) patient. During this assignment, several questions are asked on how to act in a certain situation, followed by specific automated feedback. In a final take home assignment, the healthcare provider is encouraged to plan a moment to apply the learned knowledge in clinical practice. The e-learning ends with an optional test (15 multiple choice questions; pass after ≥ 10 correct answers) after which accreditation points could be obtained (Dutch accreditation available for: ABC 1, Kwaliteitsregister V&V and Verpleegkundig Specialisten Register). Thirty five test questions were developed to provide variety when a test had to be retaken.

## E-learning optimization and evaluation

### Design

The e-learning was evaluated twice: mid-term evaluation and final evaluation. The mid-term evaluation took place directly after finishing the development of the first version of the e-learning and the collected feedback was used for optimization of the e-learning. In the final evaluation, the e-learning was re-evaluated by a new group of participants to measure if the adjustments led to improvement and to determine if the training was ready to be used in practice.

### Participants

In both evaluations, we asked healthcare providers (future users) to evaluate the e-learning. During the mid-term evaluation we additionally included placebo/communication researchers to assess the e-learning for accuracy and quality of the content. In both evaluations, participants were recruited from the professional network of the research group members, for example researchers and healthcare professionals from Leiden University Medical Center (LUMC) and Radboud University Medical Center (RadboudUMC). In the final evaluation, participants were also recruited via (social) media (e.g. on LinkedIn and in the newsletter of IVM). Healthcare providers could follow the e-learning for free and they indicated whether they agreed to use their data for research before they started. In the mid-term evaluation, placebo/communication researchers (*N* = 4) and healthcare providers (nurse *N* = 3; unknown *N* = 2) assessed the quality of the e-learning (whether the content is correct) and tested the user experience and realism of the e-learning. In the final evaluation, the e-learning was evaluated by healthcare providers (physician *N* = 5; nurse *N* = 4, other [unspecified] *N* = 9).

### Procedure & materials

In both evaluations, participants went through the e-learning by themselves, at a self-chosen moment, from their own computers. No researcher was present during this process. To evaluate the e-learning two questionnaires were designed: 1) General questionnaire and 2) Specific questionnaire. The General questionnaire, offered through the e-learning environment, included 14 questions: Five questions about the participants’ background (e.g. ‘What is your job function?’), five multiple choice questions (e.g. ‘Do you think that the e-learning is user-friendly? yes/ reasonable/not really/no’), three open ended questions (e.g. ‘How can we improve the e-learning?’), and one rating (‘What grade do you give this e-learning? scale 1–10’). Table 1 (first column) shows the multiple choice questions. The Specific questionnaire, sent by e-mail, included 14 rating questions (scale 1–10) to evaluate each separate part of the e-learning (see the first column of Table 2; e.g. ‘How would you rate the quality of the information in Module 1? 1 = very poor quality 10 = very good quality’), and one open question (‘Do you have any additional feedback?’). During the mid-term evaluation, participants completed both questionnaires. During the final evaluation, participants completed only the General questionnaire.

**Table 1:** Results e-learning evaluations General questionnaire

**Questions and answer options**	**Mid-term evaluation (N = 7)**		**Final evaluation (N = 18)**	
	**Frequency**	**Percent**	**Frequency**	**Percent**
1. Do you think that the e-learning is user friendly?				
Yes	3	43	13	72
Reasonable	4	57	5	28
Not really	0	0	0	0
No	0	0	0	0
2. Do you think the structure of the e-learning is logical?				
Yes	6	86	16	89
Reasonable	1	14	2	11
Not really	0	0	0	0
No	0	0	0	0
3. What do you think of the level of the e-learning?				
Too easy	0	0	2	11
Easy	3	43	4	22
Doable	4	57	12	67
Difficult	0	0	0	0
Too difficult	0	0	0	0
4. Can you apply what you have learned from the e-learning in daily practice?				
Yes	2	29	13	72
Reasonable	5	71	5	28
Not really	0	0	0	0
No	0	0	0	0
5. How long did it take to complete the e-learning?				
30 min	0	0	1	6
1 h	2	29	12	67
1.5 h	4	57	4	22
2 h	1	14	1	6
> 2 h	0	0	0	0

**Table 2:** Results e-learning Specific questionnaire (mid-term evaluation only)

Questions^a^	N	Mean	SD
What did you think of the …			
1. way the information was given in the introduction?	9	7.50	1
2. quality of the information in the introduction of the e-learning?	9	8.00	1
3. way the information was given in the modules?	9	8.11	0.93
4. quality of the information in module 1?	9	8.22	0.97
5. quality of the information in module 2?	9	8.44	0.73
6. quality of the information in module 3?	9	8.00	0.87
7. quality of the information in module 4?	9	7.72	0.97
8. quality of the information in module 5?	9	7.67	1.22
9. way the step-by-step case was presented?	7	8.29	0.75
10. quality of the questions in the step-by-step case?	7	7.86	0.90
11. quality of the tips given in the step-by-step case?	7	8.00	0.82
12. take home-message assignment?	8	5.88	1.64
13. way the final test was provided?	8	8.25	1.28
14. quality of the final test?	9	7.94	1.38

^a^ scale 1–10: 1 = not user-friendly/ bad quality 10 = very user-friendly/perfect quality

### VR training development and evaluation

#### Content determination

**Fig. 2:**
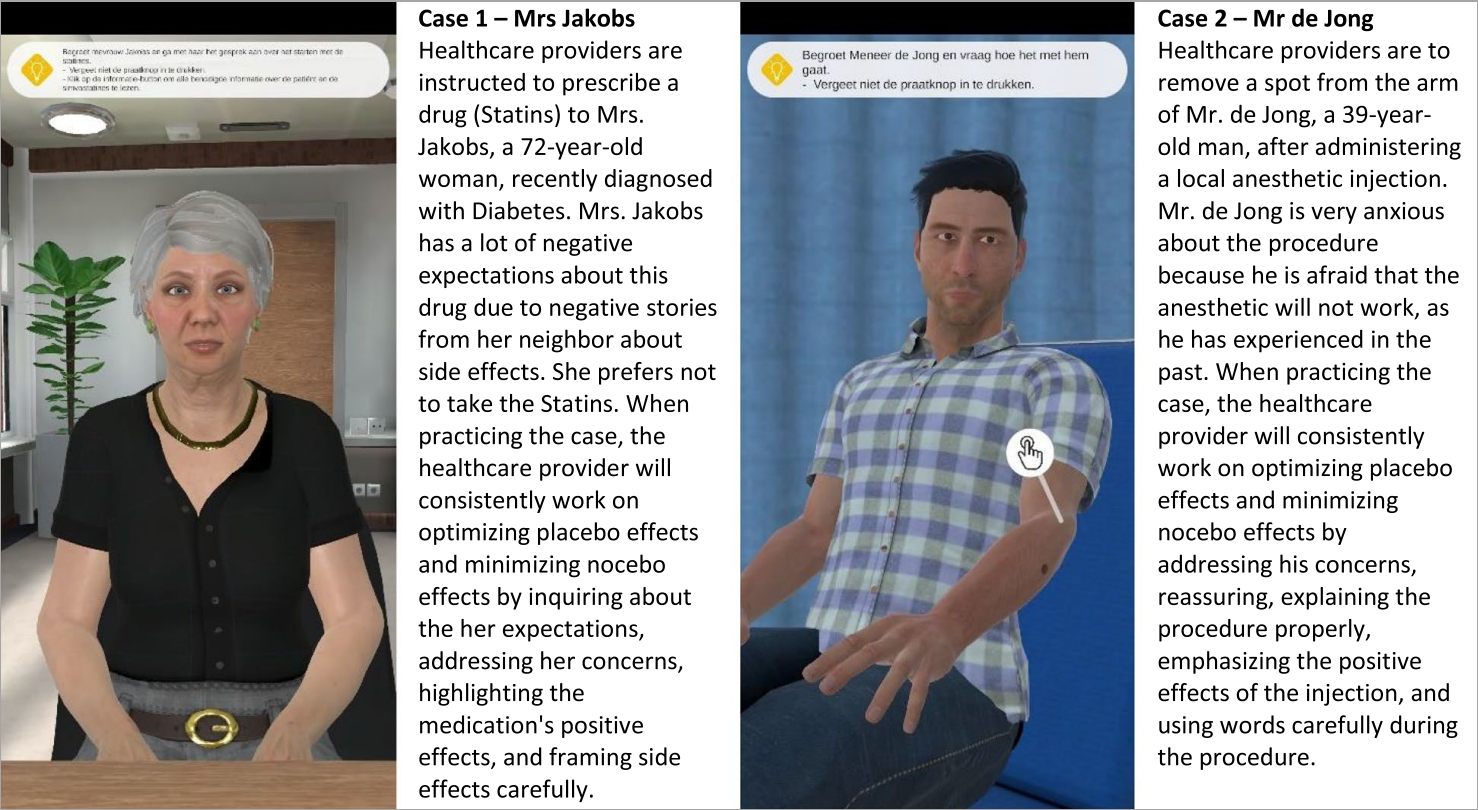
Brief description of the patient cases in the VR training

In the VR training, healthcare providers interact with simulated patients in two different scenarios while using VR headsets. The VR training focused on training those techniques that have been agreed upon by the expert group in determining the content of the e-learning, as described above. To optimize placebo effects, the provider is taught to explain why the chosen treatment is offered, to emphasize what its short- and long-term benefits are, and to display a warm and empathic attitude (e.g. by maintaining eye contact with the virtual patient). To minimize nocebo effects, the provider learns techniques such as how to identify patients at risk by recognizing negative expectancy patterns, and how to carefully introduce potential side effects of a treatment. For development of the VR training, we collaborated with The Simulation Crew (TSC). TSC is a Dutch company that specializes in developing interactive VR communication training courses using Artificial Intelligence (AI) based speech technology and simulation techniques for training and feedback. In order to ensure that the VR training fits well with conversations in clinical practice, there was structural collaboration with two clinicians (ToH and AS). During the creation of the patient cases, roleplay sessions with three nurses were conducted. Throughout the development process, intensive consultations took place between the researchers, VR developers, and involved clinicians. The researchers took into account the empirical evidence, the VR developers the developmental feasibility, and the clinicians the comparison with clinical practice. Two patient cases were designed (Fig. 2). The names within the described cases have been contrived for development of the training and do not pertain to actual individuals under any circumstances. In selecting the features of the patients, we endeavored to be as diverse as possible, by incorporating variations in gender and age.

### VR training structure

The two patient cases were integrated into an app, which can be utilized in 2D on mobile devices and in 3D with the Oculus Quest 2 VR headsets. Only the 3D version was tested in this study since the 2D version was developed later. Healthcare providers can talk aloud in the VR environment and the patient talks back. Artificial Intelligence (AI) tools, such as *speech recognition* and *natural language processing/understanding*, ensured that providers can freely interact with the patients in the VR environment and that they can explore the impact of different communication strategies on the patient. During the mid-term evaluation, the patient had a computer voice. To ensure natural responses from the virtual patients, between the mid-term and final evaluation TSC recorded all possible reactions with motion capture (gestures), facial capture (facial expression), and human voice. Moreover, the AI tracked and detected gaze direction which was used for feedback on keeping eye contact with the patient. After completing the consultation with the virtual patient, healthcare providers received personalized feedback on how they communicated with the patient, and what they could do to improve their skills.

## VR training optimization and evaluation

### Design

The VR training (3D version) was evaluated twice: during a mid-term evaluation and a final evaluation. During the mid-term evaluation, both patient cases were assessed separately because case 2 was developed after the first evaluation of case 1. During the final evaluation, both cases were re-evaluated to measure if the adjustments led to improvement and to determine if the training was ready to be used in practice.

### Participants

In both evaluations, we asked healthcare providers (future users) to evaluate the VR training. During the mid-term evaluation we additionally included placebo/communication researchers to assess the training for accuracy and quality of the content. In both evaluations, participants were recruited from the professional network of the research group members, for example researchers and healthcare professionals from Leiden University Medical Center (LUMC) and Radboud University Medical Center (RadboudUMC). During the mid-term evaluation, placebo/communication researchers (*N* = 7) and healthcare providers (physician *N* = 7, nurse *N* = 2) assessed the VR training on quality, user experience, and authenticity (i.e. to what extent the virtual conversation corresponds with a real conversation). During the final evaluation, the VR training was evaluated by healthcare providers (nurse *N* = 10; physician *N* = 8; psychologist *N* = 2; unknown *N* = 2; researcher *N* = 1). Five participants were part of both evaluations.

### Procedure & materials

Both evaluations were in person and several test days were organized in collaboration with TSC. In addition, some individual test appointments were scheduled. The procedure and materials were the same for both evaluations. Participants put on the VR headsets and went through one or both VR cases, having a conversation with the virtual patient multiple times. Participants’ interim feedback was noted by the researcher/TSC and the first impression was discussed and noted after the test. At the end of the appointment, all participants were asked to complete an evaluative questionnaire. The questionnaire contained five questions about the participants’ background (e.g. ‘What is your job function?’), multiple choice questions (e.g. ‘do you think the structure of the case is logical? Yes/Reasonable/Not really/No’), ratings (e.g. ‘how user-friendly do you find the VR training? scale 1–10’), and room for comments. See the first column of Table 3 for the multiple choice questions and ratings.

**Table 3:** Results virtual reality training evaluations

**Questions**	**Mid-term evaluation**				**Final Evaluation**			
	**Case 1**		**Case 2**		**Case 1 and 2**			
	**N**	**M; SD**	**N**	**M; SD**	**N**		**M; SD**	
1. How user-friendly do you think the VR tool is?^a^	9	7.11; 2.09	7	7.36; 1.55	23		7.17; 1.07	
2. How did you rate working with VR-glasses?^b^	9	7.22; 2.33	7	8.57; 0.53	23		7.43; 1.56	
3. What rating would you give the VR tool?^c^	9	5.94; 2.13	7	7.36; 0.48	22		6.91; 1.19	
					**Case 1**		**Case 2**	
					**N**	**M; SD**	**N**	**M; SD**
4. What did you think of the quality of the feedback?^c^	9	7.00; 1.00	7	7.14; 0.63	18	6.78; 1.44	17	6.85; 1.25
5. What rating would you give the patient case?^c^	x	x	x	x	18	6.89; 0.96	18	7.42; 1.03
	**Frequency**	**Percent**	**Frequency**	**Percent**	**Frequency**	**Percent**	**Frequency**	**Percent**
6. Do you think the structure of the case is logical?								
Yes	2	22	5	71	8	44	14	78
Reasonable	6	67	2	29	9	50	3	17
Not really	1	11	0	0	1	6	1	6
No	0	0	0	0	0	0	0	0
7. What do you think of the level of the case?								
Too easy	0	0	0	0	0	0	0	0
Easy	2	22	2	29	3	18	3	17
Doable	2	22	5	71	10	59	13	72
Difficult	5	56	0	0	4	24	2	11
Too difficult	0	0	0	0	0	0	0	0
8. Can you apply what you have learned in the case in daily practice?								
Yes	2	22	4	67	8	44	9	50
Reasonable	5	56	2	33	7	39	8	44
Not really	1	11	0	0	2	11	1	6
No	1	11	0	0	1	6	0	0

^a^ scale 1–10: 1 = not user-friendly 10 = very user-friendly

^b^ scale 1–10: 1 = very hard 10 = very easy

^c^ scale 1–10: 1 = bad quality 10 = perfect quality

## Results

### Participant characteristics

The background characteristics of all participants are summarized in Table 4.

**Table 4:** Demographic characteristics of participants

	E-learning				VR training			
	**Mid-term evaluation**		**Final evaluation**		**Mid-term evaluation**		**Final evaluation**	
	(*N* = 9)^a^		(*N* = 18)^b^		Case 1 (*N* = 9)^c^ Case 2 (*N* = 7)		Case 1 only (*N* = 5) Case 2 only (*N* = 5) Both cases (*N* = 13)	
	**Frequency**	**M; SD**			Frequency	M; SD	**Frequency**	**M; SD**
Age (years)	7	36.57;12.57			16	40.80; 12.14	23	49.65; 10.88
	**Frequency**	**Percent**	**Frequency**	**Percent**	**Frequency**	**Percent**	**Frequency**	**Percent**
Gender								
Male	0	0	3	17	6	38	8	35
Female	7	100	14	78	10	63	15	65
Other	0	0	1	6	0	0	0	0
Education								
Low*	0	0	1	6	0	0	1	4
High**	7	100	17	94	16	100	22	96
Job function^d^								
Researcher	4	57	0	0	7	44	1	4
Physician/ physician in training	0	0	5	28	7	44	8	35
Nurse/ nurse specialist	3	43	4	22	2	13	10	43
Psychologist/pedagogue	0	0	0	0	0	0	2	9
Other (unspecified)	0	0	9	50	0	0	0	0
Job Experience (years)^e^								
0-5	2	29	3	18	8	53	7	32
6-10	3	43	2	12	3	20	3	14
11-20	1	14	5	29	4	27	6	27
>20	1	14	7	41	0	0	6	27

^a^demographic characteristics data was missing for 2 participants

^b^around 100 healthcare providers followed the e-learning, 34 gave informed consent and 18 completed the questionnaire

^c^two participants tested both cases

^d^In the final evaluation of the VR training job function data was missing for 2 participants

^e^job experience data was missing for 1 participant in all evaluations except mid-term e-learning

*primary, pre-vocational and vocational

**advanced secondary and tertiary

### E-learning optimization and evaluation

#### Mid-term evaluation

**Fig. 3:**
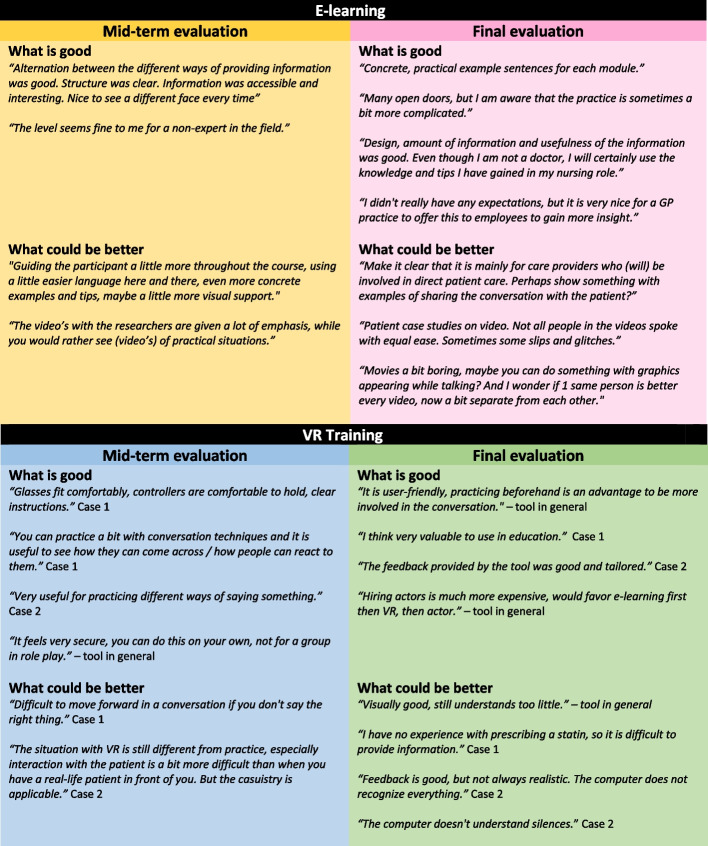
Qualitative quotes evaluation studies

During the mid-term evaluation, all components of the e-learning were rated positively (range *M* = 7.5 – *M* = 8.4) except the *take-home assignment* (*M* = 5.9, *SD* = 1.64) (Table 2). The alternation between the different types of information (e.g. text, video, assignment) was experienced as positive, as well as the structure, user-friendliness, and level of the e-learning (Table 1). The e-learning as a whole was assessed with a 7.9 (*N* = 7*, SD* = 0.90). Figure 3 shows some qualitative comments of participants per study.

#### Optimization

Based on the quantitative and qualitative analysis of the mid-term evaluation, the following adjustments were made to optimize the e-learning:

- The *take home assignment* was offered as an optional, instead of a required part of the training.
- We added a clear overview screen at the beginning of the e-learning with the aim, the structure, the welcome video and an overview of the chapters.
- More example phrases, that healthcare providers can use in daily practice, were added (e.g. how to explore expectations).
- Detailed feedback on grammar and the general layout of the e-learning was processed when possible.

## Final evaluation

The e-learning improved in terms of user-friendliness (‘yes’ from 43 to 72%) and applicability in practice (‘yes’ from 29 to 72%), see Table 1. The overall assessment was equal in both evaluation moments (*N* = 7*, M* = 7.9, *SD* = 0.90 vs. *N* = 18,* M* = 7.9, *SD* = 0.76). Quotes of participants confirmed that the added practical examples were helpful: e.g. *“Design, amount of information and usefulness of the information was good. Even though I am not a doctor, I will certainly use the knowledge and tips I have gained in my nursing role”*. Enhancing the quality of the videos or including healthcare provider-patient interaction videos are potential suggestions for improvement (see quotes in Fig. 3).

### VR training optimization and evaluation

#### Mid-term evaluation

During the mid-term evaluation, case 1 was rated less positively than case 2 (*M* = 5.9; SD = 2.13 vs.

*M* = 7.4; *SD* = 0.48). More than half of the participants scored case 1 as *difficult*, however all participants perceived case 2 as either *doable* or *easy*. In both cases, participants indicated that the interaction with the simulated patient was difficult because the tool does not always understand everything they said (due to speech recognition limitations). This resulted in a stiff and sometimes unnatural conversation flow. The user-friendliness, on the other hand, was immediately assessed as sufficient in both cases (*M* = 7.1; *SD* = 2.09 and *M* = 7.4; *SD* = 1.55, respectively), see Table 3 and Fig. 3.

#### Optimization

The first step towards VR training improvement was that all possible reactions/movements of the virtual patient were recorded by an actor in a motion-sensitive suit. This improvement gave the simulated patient a more human appearance. The following adjustments were also made to optimize the VR training:

- The recognition and vocabulary of the simulated patient was expanded, allowing the system to better understand what the participant is saying and improve the responses.
- After the participant welcomed the patient, the patient starts talking directly instead of waiting for a question from the trainee, which makes the start of the conversation smoother.
- More instructions were added to guide the participant through the conversation.
- The visuals were optimized (e.g. enhanced legibility of the computer screen in the virtual environment).

#### Final evaluation

The final evaluation showed that case 1 improved in terms of structure, level and overall rating (see Table 3). Case 2 was assessed almost equal as in the mid-term evaluation. In both cases about half of the participants perceived the acquired knowledge as directly *applicable* in clinical practice (44% and 50%, respectively), almost the other half perceived it as *reasonably applicable* (39% and 44%, respectively). The comments also indicated that the VR training was perceived as valuable: e.g. *“I think very valuable to use in education”*. For additional quotes, see Fig. 3. The VR training as a whole was assessed with a 6.9 (*N* = *22, SD* = 1.19). Instances where the avatar does not understand the participant or gives inappropriate responses remain a focus point for improvement in the future.

## Discussion

We developed and evaluated an innovative communication training, consisting of an e-learning and VR training, for healthcare providers to optimize placebo and minimize nocebo effects through healthcare provider-patient communication. Results of the evaluation studies show that both healthcare providers and communication/placebo researchers were mostly positive about the communication training. The e-learning was experienced as user-friendly and the content was perceived as accessible, interesting, and easily applicable in clinical practice. Enhancing the quality of the videos or including healthcare provider-patient interaction videos are potential suggestions for improvement. The VR training was experienced as user-friendly as well, and as offering a safe learning environment. Instances where the VR avatar does not understand the participant or gives inappropriate responses remain a focus point for improvement in the future.

The growing acknowledgement of the power of communication in healthcare is a positive development that results in an increase in communication training programs for healthcare providers. Existing communication training courses often focus on shared decision making [32], person centered care [33], or serious illness communication [34–36]. Fewer training courses focus on how to utilize placebo effects in clinical practice [37–39]. What our training adds to the existing training courses is that we focus on both optimizing placebo effects, and also minimizing nocebo effects. In addition to educating healthcare providers about the potential impact of expectations and empathy, we also train them in effectively informing patients about placebo and nocebo effects. We utilize various learning methods, including text, video, assignments, and virtual reality, and aim to be accessible to healthcare providers in all disciplines.

Setting up this e-learning and VR training presented some limitations and taught us some lessons that may also be helpful for others. First an issue, common in interdisciplinary collaborations [40], that arose at the initial stage of the development was that the researchers and educational experts (IVM and TSC) experienced lack of expertise in each other’s field. Learning each other's language was time-consuming, but frequent consultation at the beginning of the project has been helpful. The growth of knowledge of each other's field is reflected in the finding that VR case 2, which was developed after a first version of case 1 was evaluated, was immediately assessed better than case 1. Second, a well-known problem of VR is that it remains difficult to be authentic (i.e. to what extent the virtual patient reacts like a real patient) due to technical challenges [23, 40, 41]. In our VR training, we decided to use the technique *natural language processing*, instead of the more conventional *choice-based dialogue*. The use of *natural language processing* enables a real conversation with the virtual patient, however it is also more challenging and time-consuming to ensure a smooth conversation flow. Our results reveal that the authenticity did improve as we progressed in the development. More use of the VR training will improve speech recognition, due to the self-learning abilities of the applied AI. Third, during the final evaluation of the e-learning, we were not able to ascertain the specific medical roles of the participants involved, as the response option 'other' could not be elaborated upon. Fourth, the initial plan was to develop and evaluate the e-learning and the VR training simultaneously as one product. However, due to practical considerations (e.g. time constraints and the distribution of required expertise among multiple partners) separate developmental and evaluation phases were needed. Consequently, this separation led to relatively small sample sizes for all evaluations, which are a limitation of this study. Nonetheless, the separate development has also resulted in an additional benefit: the e-learning and VR training are two self-contained, full-fledged and complementary training tools. These tools can be offered independently or combined as a full training. Combining both training tools, starting with the e-learning followed by the VR training, may enhance the effectiveness of the training [35].

Development of this first-of-its-kind communication training offers opportunities for future directions. In a follow-up study the effect of this training on healthcare providers’ communication should be studied. To assess the improvement of healthcare providers' theoretical knowledge, the e-learning test can serve as a measurement instrument for both pre- and post-training evaluations. In the VR training, healthcare providers' communication is already being assessed through a scoring system, which is currently used to determine the personalized feedback. The score could potentially serve as a pre- and post-measurement, or it can be studied whether there is an enhancement in the scores when healthcare providers go through the case studies multiple times. Next, it can be investigated whether the acquired communication skills impact patient outcomes on both short- and long-term levels. Some potentially expected outcomes may include increased treatment effectiveness, higher levels of satisfaction and trust, as well as reduced anxiety and perceived side effects [18, 42–44]. Another direction for the future is translation of the training. The current training has been developed from a Dutch (East European) perspective and is only available in Dutch. Translating the training to other languages and cultures is an important next step, where cultural differences and preferences must be taken into account [45, 46]. A last valuable direction is expanding the VR training with more specific cases to connect even better with healthcare providers from all (para)medical disciplines (e.g. physiotherapists and psychologists). When developing new cases in the future, it is important to strive for diversity in patient features, such as gender, age, and culture. In future AI developments, it's essential to stay informed about ongoing advancements, potential biases, and ethical discussions.

### Availability

The e-learning and VR training (2D and 3D) are already offered in The Netherlands and available via the websites of IVM and TSC. After completing the e-learning, Dutch accreditation is available for: ABC 1, Kwaliteitsregister V&V and Verpleegkundig Specialisten Register.

**Training introduction video:** https://www.youtube.com/watch?v=3N6r_Syk2SA

**IVM:** https://www.medicijngebruik.nl/scholing/e-learning/4942/behandeleffecten-verbeteren-via-communicatie

**TSC:** https://thesimulationcrew.com/producten/placebo/

## Conclusion

To conclude, we have developed an innovative and user-friendly communication training that can be used to teach healthcare providers how to optimize placebo effects and minimize nocebo effects through healthcare provider-patient communication. The training consists of an e-learning and VR training (2D and 3D) which can be followed separately or together. Placebo/communication researchers and healthcare providers have provided a favorable evaluation of the training. However, the training’s potential effect on the communication of healthcare providers has not yet been studied. Future studies can focus on translating the training into other languages and cultures, improving the authenticity of the VR training, expanding with additional VR cases, and measuring the expected effects on healthcare provider communication skills, and subsequently, on patient outcomes.

## Data Availability

The data generated and/or analyzed during the current study will be made available upon request (corresponding author: j.westendorp@fsw.leidenuniv.nl) after publication via the DataverseNL research data repository.

## Abbreviations

VR: Virtual reality
IVM: Dutch Institute for Rational Use of Medicine (Instituut Verantwoord Medicijngebruik)
TSC: The simulation crew
